# Permissive Hypotension in a Patient with Severe Hypernatremia: A Case Report

**DOI:** 10.5811/cpcem.1422

**Published:** 2024-03-25

**Authors:** Andrea Hlady, David Kerner, Laura E. Walker

**Affiliations:** * Mayo Clinic Health System, Department of Emergency Medicine, Alberta Lea, Minnesota; † Mayo Clinic, Department of Emergency Medicine, Rochester, Minnesota

**Keywords:** *metabolic disturbance*, *hypernatremia*, *permissive hypotension*, *case report*

## Abstract

**Introduction:**

Severe hypernatremia is a critical situation, and when coupled with intravascular depletion and hypotension can create a treatment dilemma.

**Case Report:**

We present the case of a medically complex patient who had gradually worsening alteration of mental status and mean arterial pressures in the 50s on presentation to the emergency department.

**Conclusion:**

Final diagnoses included severe hypernatremia and hypovolemic shock secondary to poor oral intake. We used judicious fluid repletion with gradual improvement in sodium levels and permissive hypotension to avoid rapid osmotic shifts. Balancing reperfusion and the risk for osmotic effects of aggressive fluid resuscitation can be a challenging situation for the multidisciplinary team.

## INTRODUCTION

Mental status change in older adults is a common presenting complaint and often due to systemic processes such as infection, trauma, central nervous system (CNS) impairment, medications, metabolic derangements, cardiopulmonary dysfunction, or iatrogenic effects.^1^ Taken as a multifactorial process, risk of delirium is increased by nursing home residents’ cognitive impairment, hearing impairment, and history of stroke, with potential strong associations with frailty and malnutrition.^2^ Due to the broad differential, evaluations for delirium and mental status changes often include screening for underlying infections, metabolic derangements, dehydration, primary CNS causes, trauma, and other causes of end organ damage.

Hypernatremia, serum sodium greater than 145 milliequivalents per liter (mEq/L), is common in older adults, and nursing home residents are particularly at risk as oral rehydration can be challenging.^3^ Most cases are found at the extremes of age, when individuals are less able to act on or detect thirst. Additional causes include insensible losses, gastrointestinal losses, central or nephrogenic causes, and diuresis. Increased oral intake of sodium without concomitant free water can also result in hypernatremia. Treating both the underlying condition and restoring normal physiology underlies the approach to care in the emergency department (ED). Acute vs chronic onset of hypernatremia determines the rate at which sodium is corrected to avoid iatrogenic cerebral edema.^4^ We describe a case in which hemodynamic instability and severe hypernatremia presented together, creating a complex scenario for ED resuscitation.

We adhered to the CARE guidelines for reporting case studies.^5^

### Patient Information

A 64-year-old woman was transported to the ED via emergency medical services (EMS) with altered mental status and generalized weakness. Her medical history was significant for contractures of unknown etiology, osteoporosis, psoriatic arthritis, bullous pemphigoid, paroxysmal supraventricular tachycardia, hypertension, and mild cognitive impairment. She required ongoing nursing care due to her limited mobility caused by her contractures.

Upon ED arrival she was unable to contribute to the history secondary to her mental status changes. Family informed the ED team that she had been unwell for the prior three days with decreased oral intake and increased confusion. They had noticed that she was not eating at her recent birthday celebration and that her speech seemed “off” when speaking via telephone. On the day of presentation she had not called her family and, therefore, a family member requested that a nurse check in on her. They also visited the patient later that day finding her confused, prompting activation of EMS.

### Clinical Findings

On arrival to the ED her blood pressure was initially measured at 101/64 millimeters of mercury (mm Hg) but within 30 minutes was 75/63 mm Hg, heart rate 137 beats per minute, respiratory rate 34 breaths per minute with normal saturations of 97%, and a temperature of 36.6° Celsius (C). Her weight was 35.3 kilograms. She was confused and unable to answer questions or follow commands. She appeared cachectic, with bitemporal wasting. Her eyes were sunken, and there was purulent drainage from the right eye. Mucous membranes were dry. Her eyes were open, she was moaning with incomprehensible speech, and she withdrew from pain. She had weak peripheral pulses. Cardiopulmonary exam revealed tachycardia and clear lungs bilaterally. Her abdomen was soft, although she did appear to have voluntary guarding. She had severe contractions of the upper and lower extremities. Her skin was warm and dry.

With her hemodynamic abnormalities the priority after primary survey was to obtain intravenous (IV) access. This was impaired by the diffuse contractures as well as hypovolemia. Ultrasound-guided peripheral IV and central venous catheter access were attempted, but veins appeared to be non-compressible and concerning for diffuse clot burden precluding additional attempts. Intraosseous access was obtained, and the patient was given a bolus of one liter normal saline and started on lactated Ringer’s at a rate of 200 milliliters per hour (mL/hr). A timeline of patient care is presented in the Table 1.

**Table 1.** A timeline of the patient episode of care.

Elapsed Time	Event
00:00	Patient arrival
00:12	Initial physician evaluation
00:18	Attempts at peripheral access unsuccessful HR: 137; BP: 101/64
00:29	1 liter 0.9% normal saline bolus initiated
00:44	Point-of-care glucose 87 mg/dL
	Labs drawn
00:48	HR: 136; BP: 75/63
00:53	Complete blood count resulted
01:07	Lactate resulted
01:16	Basic metabolic panel resulted
01:18	HR: 136; BP: 75/65
01:48	HR: 140; BP: 75/58
01:51	Urinalysis resulted Family present to provide additional history
1:58	Intraosseous needle placed
02:00	HR: 140; BP: 114/98
02:29	Bedside echocardiogram: hyperdynamic myocardium, collapsed IVC noted
02:48	HR: 116
02:50	Urine osmolality resulted
03:03	HR: 131; BP: 73/55
03:24	Peripheral IV access obtained
04:31	Point-of-care venous blood gas obtained, repeat lactate improving
04:33	Pipercillin/sulbactam administered, lactated Ringer’s initiated at 200 mL/hour HR: 122; BP: 73/46
04:47	Arterial line placed
04:48	HR 118; BP: 73/47
05:03	HR 114; BP: 76/48
05:18	Heparin drip initiated due to concern for clot HR: 119; BP: 71/43
05:21	Imaging results reassuring, decision to admit to medical intensive care unit
05:48	HR: 122; BP: 83/51
06:03	HR: 122; NP: 81/49 Patient transported to intensive care unit
8:00–30:00	D5W administered at varying rates BP (MAP) ranges from 55-69
86:00	Sodium level 145 mEql/L

*HR*, heart rate; *BP*, blood pressure; *IVC*, inferior vena cava; *IV, intravenous; mL, milliliters*; *D5W*, dextrose 5% in water; *MAP*, mean arterial pressure; *mg/dL*, milligrams per deciliter; *mEq/L*, milliequivalents per liter.

### Diagnostic Assessment

Lab studies included electrolytes, complete blood count, lactate, coagulation profile, blood cultures, and COVID-19 and influenza swabs, as well as urinalysis. Significant findings are shown in the Table 2. Upon discovery that her sodium was 176 mEq/L, fluid resuscitation was paused to determine appropriate rate of administration for safe correction.

**Table 2.** Notable lab studies.

WBC	27.4 × 10^9^/L
Neutrophil count	21.89 cells/L
Hematocrit	47.6%
Sodium	176 mEq/L
Chloride	131 mEq/L
Creatinine	2.85 mg/dL (baseline 0.7)
BUN	85 mg/dL
INR	1.8
Lactate	3.7 mEql/L
Urine	Red appearance, cloudy; + RBCs, + WBCs, + ketones, + nitrites, + leukocyte esterase

*WBC*, white blood cells; *BUN*, blood urea nitrogen; *INR*, international normalized ratio; *RBC*, red blood cells; mEq/L, milliequivalents per liter; *mg/dL*, milligrams per deciliter.

Her hypotension, apparent abdominal discomfort, and concern for diffuse clotting prompted several imaging studies. These were delayed secondary to difficulty obtaining IV access and concern for potential significant clot burden. Ultimately when they were obtained computed tomography (CT) of the abdomen and pelvis demonstrated no acute findings, CT of the brain showed no apparent abnormalities, and lower extremity ultrasound did not reveal acute deep vein thrombosis.

The patient continued to have tachycardia and hypotension following the initial liter of crystalloid provided. The discovery of profound hypernatremia prompted a treatment dilemma, in which slow correction of sodium was pursued rather than rapid correction of hemodynamics. Our assessment, based on the history provided by the family, was that this was likely a chronic development of hypernatremia. Clinically, she appeared dehydrated and in need of additional fluids. In addition to dehydration and resulting hypernatremia, we considered the possibility of sepsis with a possible urinary source. Her elevated lactate, creatinine, and urinary ketones, as well as her hemodynamics, were evidence of a need for additional fluids. The degree of sodium elevation she had is associated with a risk of mortality of 75%^6^; additionally, her elevated lactate in the setting of infection contributed additional risk of mortality within 28 days.^7^

### Therapeutic Intervention

After the initial one liter of normal saline was given and we discovered her hypernatremia, we adjusted fluid administration to achieve a goal rate of sodium correction between 10–12 mEq/L per day, starting with lactated Ringer’s at 200 cc/hr. An ED pharmacist assisted to ensure appropriate therapy. She was treated empirically for possible urosepsis with piperacillin-sulbactam (2.25 g) pending culture results.

During her ED stay our team struggled with the conflicting desires to treat her hypotension and tachycardia and provide resuscitation for her sepsis/hypovolemia while managing her hypernatremia. Aggressive rehydration would have likely resulted in cerebral edema and a very poor outcome. We opted to pursue permissive hypotension in the ED, and this course of care was continued into the intensive care unit (ICU). Upon ICU admission, she was started on dextrose 5% in water at a rate of 100 milliliters per hour (mL/hr) with additional free water administered via nasogastric tube at 50 mL/hr.

### Follow-up and Outcomes

During hospitalization, nutritional studies revealed severe malnutrition. Additional history at that time uncovered she had over 20% weight loss over the course of the prior year. She was transferred to the regular medical floor when her sodium reached 152 mEq/mL, two days after admission. Her hospital course was characterized by difficulty with oral intake. A palliative care consult was performed, and a family decision was made to pursue feeding via nasogastric tube followed by percutaneous endogastric tube. Anemia developed following rehydration, thought to also be associated with frequent lab draws, and she was transfused with one unit of packed red blood cells. She had episodes of aspiration with resulting tachycardia and tachypnea. Imaging studies revealed an exudative pleural effusion with negative cultures. Her symptoms improved without antibiotics. She remained stable for the remainder of her hospitalization and was discharged on hospital day 24 to a skilled nursing facility with parenteral feeding. Figure 1 demonstrates the rate of sodium correction along with representative mean arterial pressure (MAP) during this period.

**Figure.**
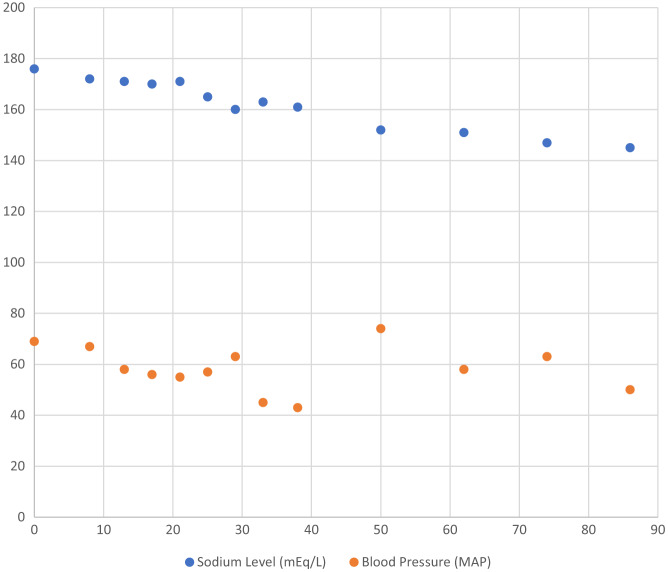
Hourly trends of sodium level and mean arterial pressure. *MAP*, mean arterial pressure; mEq/dL, milliequivalents per deciliter.

## DISCUSSION

Management of hypernatremia is a “bread and butter” topic in emergency medicine. However, the role of permissive hypotension in resuscitation is most often cited in reference to patients with trauma and severe hemorrhage,^8^ ruptured abdominal aortic aneurysm,^9^ and vasodilatory shock.^10^ Neither at the time care was provided, nor subsequently, did we identify literature discussing the tenuous resuscitation of patients with severe hypovolemia and hypotension in the setting of profound hypernatremia.

The immediate care for this patient presented a situation that was stressful for the ED team. Persistent hypotension and tachycardia in the ED were noted by team members including nursing, physician trainees, and pharmacists. This was remediated by good team communication and collaboration. A shared understanding of the order of operations we would undertake and the reasoning behind permissive hypotension was crucial to providing appropriate care for the patient. When we are pushed out of our typical algorithm of “airway, breathing, circulation” and instead need to tend to long-term survivability due to potential metabolic/neurologic compromise, the smooth care we typically aim to provide is disrupted. Knowing when and how to go “off protocol” from the basic approach to resuscitation is a key skill in emergency medicine.

The goal of treating hypotension is to reduce end organ injury. This is primarily achieved through fluid resuscitation and vasopressors. However, there are times when either fluids or vasopressors may be detrimental. Consider aggressive fluid resuscitation in pulmonary embolism or hypernatremia, crystalloid resuscitation in hemorrhagic shock, or high vasopressor use and tissue hypoxia as well as increased mortality in vasodilatory and hemorrhagic shock.^8^^,^^11^^–^^15^ In these instances, permissive hypotension may be in the best interest of the patient, but what are the effects of long-term controlled permissive hypotension and what degree of hypotension should we allow?

There are very few studies discussing this issue and even fewer that can direct our practice. Most studies are focused on the effects of hemorrhagic shock. A retrospective cohort study of ICU patients suggests that the MAP at which terminal cardiovascular collapse occurs is somewhere between 30–46 mm Hg^15^; and we are taught that 65 mm Hg is the optimal MAP goal, and in some instances higher (eg, post-operative spinal cord perfusion). In a randomized control trial evaluating ICU patients ≥65 years comparing permissive hypotension to usual care, they did not find a statistically significant difference in 90-day mortality and no difference in serious adverse events. Most adverse events related to acute renal failure and supraventricular cardiac arrythmias. Cognitive decline and health-related quality of life at 90 days and at one year were similar between the two groups.^10^ Allowing permissive hypotension and deferring to other priorities in resuscitation may offer an advantage with minimal downside in cases such as severe hypernatremia and volume depletion.

The primary take-aways from this case include the importance of rapid reassessment and pivoting from aggressive crystalloid resuscitation when hypernatremia was identified; and the use of permissive hypotension in hypovolemic hypernatremia to prioritize central nervous system stability and establish a shared understanding of the treatment priorities for ED team members. We believe that ED and subsequent ICU care were essential to the patient’s survival to discharge.
